# An Improved Transwell Design for Microelectrode Ion-Flux Measurements

**DOI:** 10.3390/mi12030273

**Published:** 2021-03-06

**Authors:** Boris Buchroithner, Pavel Spurný, Sandra Mayr, Johannes Heitz, Dmitry Sivun, Jaroslaw Jacak, Jost Ludwig

**Affiliations:** 1 School of Medical Engineering and Applied Social Sciences, University of Applied Sciences Upper Austria, Garnisonstr. 21, 4020 Linz, Austria; Boris.Buchroithner@fh-linz.at (B.B.); sandra.mayr@fh-linz.at (S.M.); jaroslaw.jacak@fh-linz.at (J.J.); 2 Institute of Microbiology of the Czech Academy of Sciences, Zamek 136, 37333 Nove Hrady, Czech Republic; spurny@nh.cas.cz (P.S.); ludwig@nh.cas.cz (J.L.); 3 Institute of Applied Physics, Johannes Kepler University Linz, Altenberger Str. 69, 4040 Linz, Austria; johannes.heitz@jku.at

**Keywords:** multiphoton lithography, biocompatible polymers, BisSR, HUVECs, MIFE, ion flux

## Abstract

The microelectrode ion flux estimation (MIFE) is a powerful, non-invasive electrophysiological method for cellular membrane transport studies. Usually, the MIFE measurements are performed in a tissue culture dish or directly with tissues (roots, parts of the plants, and cell tissues). Here, we present a transwell system that allows for MIFE measurements on a cell monolayer. We introduce a measurement window in the transwell insert membrane, which provides direct access for the cells to the media in the upper and lower compartment of the transwell system and allows direct cell-to-cell contact coculture. Three-dimensional multiphoton lithography (MPL) was used to construct a 3D grid structure for cell support in the measurement window. The optimal polymer grid constant was found for implementation in transwell MIFE measurements. We showed that human umbilical vein endothelial cells (HUVECs) efficiently grow and maintain their physiological response on top of the polymer structures.

## 1. Introduction

The transwell migration/invasion and the spreading assay are used to analyze the ability of single cells to directionally respond to chemoattractants and treatments and to test cell capacity to adhere to the extracellular matrix, respectively. The classic transwell migration detection system uses a hollow plastic chamber sealed at one side with a porous membrane. This chamber is suspended over a larger well, which contains medium and/or chemoattractants. Cells are placed inside the chamber and allowed to migrate/spread through the pores to the other side of the membrane. Migratory cells are stained and counted. The assay was used to analyze the response of cells to chemokines or growth factors and key regulatory factors that affect cell migration [1]. Thayanithy et al. have used a transwell assay to test the effects of intercellular transfer of a therapeutic oncolytic virus and viral-activated drugs between cells via tunneling nanotubes [2]. The assay was also used to analyze the invasive abilities of tumor cells [3] and for detection and enumeration of circulating tumor cells based on their invasive property [4]. Most of these studies have used optical (i.e., fluorescence) methods.

Besides optical, electrochemical methods are used for analysis of the cellular processes inside the transwell system. To achieve quantitative real-time monitoring, optical methods can be parallelized in combination with impedance spectroscopy. The impedimetric analysis of covered electrodes and the relative impedance maximum values revealed extended information regarding the contribution of proliferative effects [5]. More advanced systems use organic electrochemical transistors fabricated on a flexible substrate in transwell support to monitor the global transport of chemicals like H_2_O_2_ [6].

For a more local and precise analysis of electrophysiological processes, such as membrane transport activity, which is crucial for understanding homeostasis, maintenance of cell osmotic potential, nutrient acquisition, and signaling of cells, ion-sensitive methods are used. A number of methods have been employed to measure the transport of ions in and out of the cell such as nuclear magnetic resonance (NMR) spectroscopy, ion imaging by fluorescence microscopy (FM), patch-clamp, and microelectrode based flux estimation (MIFE) [7]. While NMR and FM have significant drawbacks (not every nutrient can be studied and interaction of ion probes with cell metabolism [7]), patch-clamp allows local and sensitive (pA range) measurements of ion currents [8,9]. MIFE is a non-invasive alternative to patch-clamp, which can measure ionic currents across whole cells, layers of cells, or tissues [10]. MIFE is based on ion-selective microelectrodes, which are moved between two positions (closer and further) from the area of interest to measure the local electrochemical potential (easily convertible to ion concentration) of a specific ion. Ion fluxes are calculated from the small differences of concentrations between these two positions. Besides, various ions such as K^+^, Na^+^, Cl^-^, H^+^, or Ca^2+^ can be measured simultaneously. So far, MIFE was mainly used for measuring ion currents across membrane of plant tissues [11], monolayers of unicellular micro-organisms [12], and monolayers of animal cells [13,14].

Multiphoton lithography (MPL) is a well-known nano/microfabrication tool [15], [16], which is suitable for 3D structuring with lateral and axial resolutions 200 nm and 500 nm, respectively [17]. Compared to alternative fabrication techniques such as nanoimprint or electrospinning lithography, MPL gives full flexibility to structured geometry and is much faster and cheaper than electron/ion beam lithography techniques.

In this work, we altered commercially available transwell inserts by exchanging the standard membrane to a non-permeable foil modified with a multiphoton lithography written structure inside a laser-drilled hole [18]. The improved membrane significantly increases the interaction area of cells with an environment. Large free-space (interaction) area and small membrane thickness allows for direct coculture, which is substantial for reproducible biological models [19,20]. This transwell insert was tested via seeding with HUVECs. First, we used MIFE to test the bioinertness of the material by cultivating HUVECs on coverslips with 2D MPL structures. Subsequent MIFE measurements inside the modified transwells showed that parameters of cell-supporting polymer grids directly influence the cell layer quality and, therefore, the ion flux measurements. Finally, we showed that MIFE could be used inside the transwell systems to locally characterize the physiological processes in cell monolayers.

## 2. Materials and Methods

### 2.1. Lithography System

All laser cutting and lithography were done by a custom multiphoton lithography system (Workshop of Photonics (WOP), Vilnius, Lithuania), which includes an ultra-short pulsed laser (1 MHz repetition rate, 290 fs pulse duration, CARBIDE, Light Conversion, Vilnius, Lithuania) operated at 1030 nm and 515 nm wavelength (first and second harmonic, respectively). Sample motion was performed by a 3-axis stage (Aerotech Inc., Pittsburgh, PA, USA). A galvanometric mirror scanner (Aerotech Inc., Pittsburgh, PA, USA) was used for faster lateral (XY) scanning/writing.

### 2.2. Resin Formulation

For two-photon lithography, biocompatible photoresist BisSR was used [21], which is a mixture of 29% of Bis-GMA (Esschem Europe, Seaham, UK) and 71% ethoxylated bisphenol A dimethacrylate (SR348C) (Sartomer, Colombes, France). All monomers are used as received and stirred with a magnetic stirrer until homogeneously mixed. As the photoinitiator 1 wt % of IC819 was used.

### 2.3. Fabrication of Grid on Coverslides

Two-dimensional grids were fabricated out of BisSR photoresist by second harmonic (515 nm) CARBIDE laser. Writing was performed in dip-in configuration with a 63× 1.25NA objective (Zeiss, Oberkochen, Germany). The overall size of the grid was 1 mm × 1 mm with a grid constant of 10 µm.

### 2.4. Fabrication of the Modified Transwell Inserts

**Figure 1:**
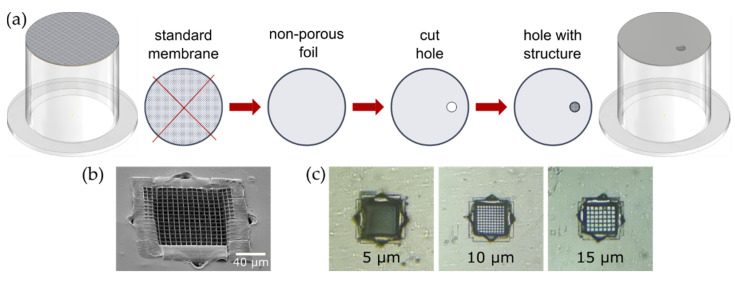
(**a**) Schematic representation of the transwell modification. The standard membrane is cut from a transwell insert; a non-porous, non-permeable foil is attached, in which a hole is cut by the laser; a grid is structured inside the hole by means of multiphoton lithography (MPL). (**b**) Scanning electron microscopy (SEM) image of the polymer grid structured inside the hole. (**c**) Bright-field microscopy images of grids with grid constants of 5 µm, 10 µm, and 15 µm.

Transwell inserts (CLS3422, Corning, New York, NY, USA) were used to establish two separated compartments for the electrophysiological measurements. Therefore, the existing porous membrane of the insert was removed. To fix the modified membrane at the same position, the plastic tube is coated with a small amount of PDMS (Silgard 184, ratio 1:9, mixed and vacuumed according to the manufacturer’s specifications) and stamped onto the 1.5 cm × 1.5 cm square piece of PET-foil with a cell supporting structure. The cell supporting structure is positioned about 1 mm away from the polymer boundary to make it easily accessible for the measuring electrode. After overnight curing of the PDMS at room temperature, the protruding parts of the fixed PET-foil are cut with a scissor, taking care not to damage the tight junction. Defects can easily be closed from the outside with a second layer of PDMS. The sketch of transwell insert modification is shown in Figure 1a.

Modified membranes were fabricated in a two-step process. First, a hole was drilled into a biocompatible PET foil (biaxially-oriented polyethylene terephthalate foil, Bleher) by the first harmonic (1030 nm) of an ultra-short pulsed laser focused using a 50× air objective (NA = 0.42, Mitutoyo, Kawasaki, Japan). The laser focal spot was moved two times along the trace with a spacing of 2.5 µm (1.65 TW/cm^2^ and 1.85 TW/cm^2^ for inner and outer cutting edge side, respectively). If necessary, the inner discarded part was removed mechanically by means of an ultrasonic bath.

In the second step, the hole in the PET foil was closed with a cell supporting grid. A droplet of BisSR was drop cast onto the hole and writing carried out in dip-in configuration by 63× immersion objective (NA = 1.25, Zeiss, Oberkochen, Germany). Writing is done by the second harmonic (515 nm) of the CARBIDE laser. To improve the attachment between the 2D nanostructure and foil, a frame (35 µm wide and 30 µm thick) was written into the hole. Thereafter, the hole was closed with a 2D grid consisting of quadratic squares with a variable grid constant and a commissioned height of 2 µm. Each square was double-exposed, making the resulting bar thickness depending on the used writing speed of 200 µm/s and writing peak intensity of 1.44 TW/cm^2^. The overall size of the free-standing grid was 105 µm × 105 µm with a grid constant 5, 10, and 15 µm (Figure 1b,c).

### 2.5. Cell Cultivation and Preparation for Experiments

Endothelial cells immortalized with hTERT (HUVEC-tert) were grown in cultivation flasks [22], washed with PBS and detached by trypsin (1 mL of 10% Trypsin-EDTA solution diluted in PBS). Approximately 100,000 cells were seeded on each sample (coverslip with/without polymer structure or transwell insert), which was coated by triple-coat-mix (50 µg/mL fibronectin, 30 µg/mL collagen I, and 0.1% gelatin (#G1393, Merck, Darmstadt, Germany) in PBS for 24 h at 37 °C). Cells were grown for 2–3 days at 37 °C, with 5% CO_2_. Immediately before the experiments, samples were transferred to the MIFE system. Cultivation medium was exchanged by measurement solution (HEPES/Tris pH 7.45, ChCl 130 mmol/L, glucose 1%, CaCl_2_ 1 mmol/L, MgCl_2_ 0.5 mmol/L, KCl 0.1 mmol/L, and NaCl 0.1 mmol/L).

### 2.6. Measurement Preparation

Microelectrodes were made by pulling (pipette puller PIP 6, HEKA, Lambrecht, Germany) from glass capillaries (Ø1.5 mm, Hilgenberg, Malsfeld, Germany), baked at 210 °C (LE040K1RN, Nabertherm, Lilienthal, Germany), and silanized (Tributylchlorosilane, Fluka, Buchs, Switzerland) for achieving appropriate shape and hydrophobic surface. The tip diameter was adjusted to approximately 10 μm. As the inner (backfilling) solution, 200 mM KCl was used. The electrode tip was filled with a liquid ion exchanger (LIX) (potassium ionophore I—Cocktail A, FLUKA: for K^+^-flux measurements). As a reference electrode, a pipette with tip diameter of about 100 µm filed with 1 M NaCl in 2% agarose was used. A chlorided silver wire was inserted in both electrodes and connected with the MIFE preamplifier.

Before each experiment, calibration was carried out (six calibration points in the range between 10 μM and 3 mM of the respective ion). Calibrations were performed in tissue culture dishes (Ø35 mm, Sarstedt, Nümbrecht, Deutschland) filled with 2 mL of calibration solution (measurement solution without KCl).

### 2.7. MIFE Measurements Protocol

The sample with cells (structure on coverslip, coverslip only, and transwell insert) was removed from the incubator and transferred into a tissue culture (TC) dish with washing solution to get rid of the residual medium. Then, it was transferred to another TC dish with measurement solution.

**Figure 2:**
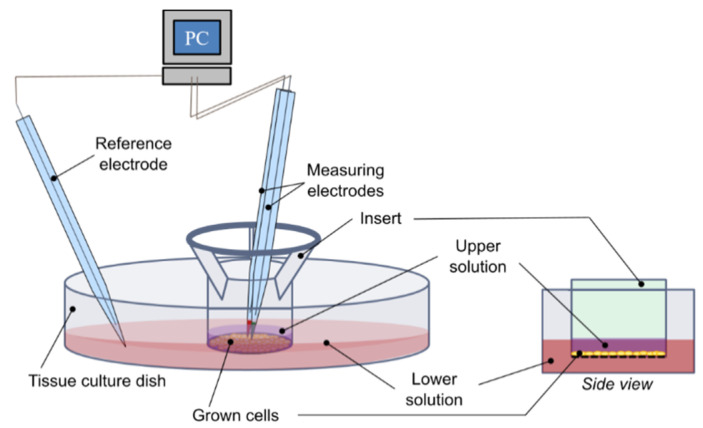
Scheme of the microelectrode based flux estimation (MIFE) experimental setup. Modified inserts with grown cells are placed into a culture dish. Ion-selective microelectrode moves between two positions, closer and further away from the cell monolayer and potentials at each position are recorded. From the potential differences, concentration differences and the flux of the specific ion are calculated.

The dish with the sample was placed in a holder and microelectrodes were dipped into the measuring solution. The reference electrode was placed near the edge of the dish, away from the sample. The measuring microelectrode was positioned approximately 10 µm above the cell layer in the center of the sample (Figure 2). During experiment, the measuring microelectrode was moved axially (up and down) with a frequency of 0.0667 Hz (movement every 15 s) and by a distance of 125 μm. The average potential of the last 12 s in each position was used to calculate the ion concentration.

**Figure 3:**
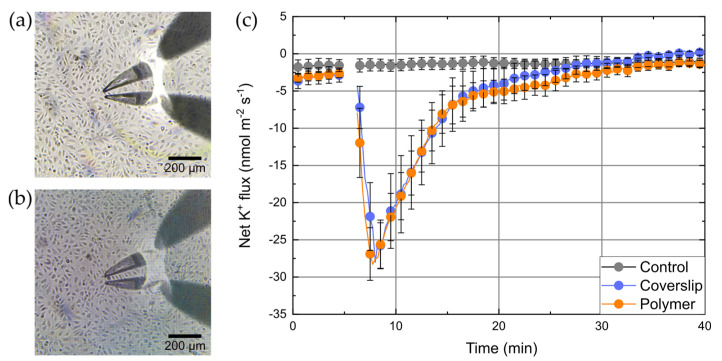
Two ion-selective electrodes situated above human umbilical vein epithelial cells (HUVECs) grown on a (**a**) coverslip and (**b**) on a polymer grid fixed on a coverslip. The grid was made of BisSR and has a grid constant of 10 µm. (**c**) Response of HUVECs to calcium ionophore (A23187). Solvent (ethanol, grey circles), or A23187 (to 10 µM, orange, and blue circles) was added after 5 min to either cells grown on a coverslip (blue circles) or polymer structures (orange circles). Data are given as mean ± SEM, *n* = 5.

Experiments were performed at room temperature (22–23 °C). First 5 min of every measurement were used for ion fluxes equilibration. When fluxes were constant (2.5 ± 1.5 nmol⋅m^−2^⋅s^−1^, see Figure 3c), the calcium ionophore A23187 (Calimycin, Merck, Darmstadt, Germany, from a 5 mM stock solution in ethanol) was added to a final concentration of 10 μM and fluxes were recorded for another 35 min. As the control measurement, ethanol at the same concentration was used.

## 3. Results and Discussion

A standard transwell system was modified by changing the default membrane to a custom-fabricated isolating membrane, which contains a measuring window covered by a 3D grid-like polymer structure. This measuring window should allow direct cell-to-cell coculture inside the transwell system. On the other hand, the polymer structure should neither affect the physiological responses of the cells and their growth, nor influence the electrophysiological measurements itself. To test the influence of polymers on cell growth and formation of monolayers, cells grown on blank coverslips or polymer structures were inspected microscopically (Figure 3a,b). We did not observe any difference in the number of cells or their appearance/shape for both substrates.

For functional testing of the integrity of cells, we took advantage of the fact that HUVECs possess high-conductance Ca^2+^ activated K^+^ channels (BK channel, K_Ca1.1_; [23,24]). Therefore, K^+^ fluxes were measured with HUVECs grown either on blank coverslips or polymer structures. Without any interventions a stable background K^+^ efflux of about −2.5 ± 1.5 nmol⋅m^−2^⋅s^−1^ was observed. BK channels were then activated by the Ca^2+^ ionophore A23187 [25] leading to an increase of intracellular Ca^2+^. In control experiments the solvent (ethanol) was added to the same concentration. The results of these measurements are shown in Figure 3c. The addition of A23187 to a final concentration of 10 µM led to a rapid increase of K^+^ efflux (within 3 min after addition) reaching peak values of about −28 ± 3.5 nmol⋅m^−2^⋅s^−1^. After that, efflux decreased and finally ceased after 30 min. K^+^ fluxes for both, cells on blank coverslips and on polymer structures show an almost identical response to A23187. In contrast, the addition of ethanol did not lead to any change in the K^+^ flux, which remained constant on its background level for the whole time of the experiment.

**Figure 4:**
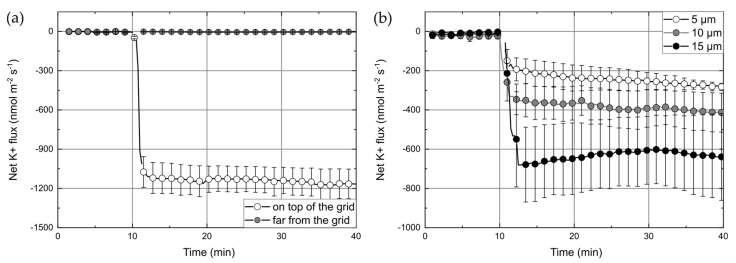
(**a**) Net K^+^ flux measured in the modified insert (10 µm grids) without cells. The flux was measured at two different positions, above the grid (white circles) and 1 mm away from the grid (gray circles). (**b**) Net K^+^ flux measured through the HUVEC-tert in the modified insert for three different grid constants (5 µm-white circles, 10 µm-gray circles, and 15 µm-black circles). Data are given as mean ± SEM, *n* = 3. KCl was added to lower compartment at t = 10 min to a final concentration of 5 mM.

The apparent ion fluxes per membrane area, which are calculated from MIFE measurements, were only correct, if the cells were fully confluent at the measurement area and reciprocally correlated to the degree of confluence. The identical apparent fluxes for cells on coverslips and polymer structures indirectly confirmed the bioinertness of the used polymers (i.e., that the polymer did not influence growth of the cells). The result also indicates that the BisSR polymer itself did not influence the K^+^ ion flux as some types of plastics, glasses, and other cell supporting surfaces can do [7]. Thereafter, we designed the transwell system setup, where the default membrane of the insert was exchanged by the custom made membrane, which is detailed described in Section 2.4. To confirm that our custom-made transwell inserts were not leaky (i.e., ions cannot diffuse between lower and upper compartments through the non-porous PET-foil except the hole with structure), MIFE (diffusion) flux measurements were carried out using inserts without cells (i) at the position of the grid and (ii) at a region 1 mm laterally away from the grid (Figure 4a). After addition of KCl (final c = 5 mM) to the lower compartment, K^+^ fluxes of −1139 ± 22 nmol∙m^−2^∙s^−1^ were observed above the grid whereas only very small fluxes (−4.2 ± 0.7 nmol∙m^−2^∙s^−1^) were measured at the position away from the grid.

Another parameter that is highly important for 3D free-hanging cells supporting structures is the grid constant. Finding a grid constant that promotes the confluent formation of the cells layer is essential for correct MIFE measurements. We addressed that question by using modified transwell inserts with grids constants of 5, 10, and 15 µm, growing HUVEC-tert cells on them and performing leak K^+^ ion flux measurements. Figure 4b shows the K^+^ fluxes measured through the HUVEC layers on the modified inserts with different grid constants. The overall behavior of the K^+^ flux was similar for all grids. After adding KCl to the lower solution, there was a rapid increase of efflux that reached saturation after 3 min. The saturation levels were dependent on the grid constant and increased with grid periodicity. The samples with 5 µm, 10 µm, and 15 µm grids showed average fluxes of about −248 ± 21 nmol∙m^−2^∙s^−1^, −385 ± 18 nmol∙m^−2^∙s^−1^, and −632 ± 21 nmol∙m^−2^∙s^−1^ respectively. Higher leak fluxes can be expected through grids with higher constants because the open area increases with the grid constant. However, this increase would be much less than the one that was observed. Therefore, it can be concluded that a decrease of fluxes with lower grid periodicity is due to a higher density/confluency of cells on a grid and therefore, a smaller flux indicates better cell coverage. The results suggest that cell-coverage of a 5 µm grids is the best one. The coverage of grids with higher constants (10 µm and 15 µm) either did not promote enough cell growth or did not allow cells to create a confluent layer. Considering that the average diameter of HUVECs was about 20–30 µm and that cells preferably started their growth from the bars, the grids with wider free-spaces (higher grid constants) most probably have the smaller initial cell density, which consequently could influence the tightness of the cell junctions. We also observed relatively high variability of flux between samples with the same grid periodicity. The uncertainty of measured fluxes (at the middle of the experiment—25 min) was ±58 nmol∙m^−2^∙s^−1^, ±96 nmol∙m^−2^∙s^−1^, and ±176 nmol∙m^−2^∙s^−1^—for 5 µm, 10 µm, and 15 µm grids respectively (Figure 4b). However, it is not clear what a source of such deviation is. Fabrication tolerances could be a reason, but for used structures dimensions (bar thickness of about 1 µm and space between bars larger than 5 µm), the MPL is an adequately precise fabrication technique. Therefore we assumed that other experimental variables, such as reproducibility of the cell culture and the accuracy of the chemoattractants addition, could have influenced measurements.

## 4. Conclusion

We showed the design and fabrication of a new type of insert for transwell systems. The new design allows an investigation of physiological processes in direct cell-to-cell coculture, which could give deeper insight into cell–cell contact-dependent membrane transport processes and thus allow one to perform experiments in conditions closer to in-vivo. Besides, the proposed design allows simultaneous ion-flux (MIFE) and fluorescence microscopy measurements—if an appropriate distance between bottom of the insert and the tissue culture dish is used. As an addition, the finite size of the measuring window relaxes requirements for the cell layer confluence. We showed that HUVECs efficiently grow and keep their functional physiological response on 2D polymer grids. The used biocompatible polymer (BisSR) did not modulate the K^+^ fluxes and thus most likely did not interfere with physiological processes. However, further experiments are needed to analyze the possible influence of BisSR on fluxes of other ions (e.g., Na^+^, Ca^2+^, and H^+^) and other physiological parameters. K^+^-fluxes across presumed “monolayers” of HUVECs were tested using grids with different periodicity constants. It was found that smaller grid sizes led to smaller translayer fluxes. A possible explanation for this observation is that with these grids, higher cells confluency/density was obtained. The standard transwell insert has a membrane with 8 µm pores. We implemented grids with smaller and larger periodicity constants. The result suggests that a smaller grid constant is preferable for obtaining a reproducible HUVEC cell layer. However, it should be mentioned that the results are only valid for the HUVECs used in this experiment. A systematic study of structures with even smaller grid constants and different cell types would be an appropriate step to generalize this finding. We believe that the developed design of the transwell system, where direct cell-to-cell coculture and simultaneous optical and physiological methods (MIFE) are possible, will be useful for investigation of cells membrane transport activities in an environment close to in-vivo conditions.

## Data Availability

The data presented in this study are available on request from the corresponding author.
